# Link between Motor Competence and Health Related Fitness in Children and Adolescents

**DOI:** 10.3390/sports5020041

**Published:** 2017-06-15

**Authors:** Carlos Luz, Rita Cordovil, Gabriela Almeida, Luis P. Rodrigues

**Affiliations:** 1 Faculdade de Motricidade Humana, Universidade de Lisboa, 1495-688 Lisboa, Portugal; 2 Escola Superior de Educação de Lisboa, Instituto Politécnico de Lisboa, 1549-003 Lisboa, Portugal; 3 Laboratory of Motor Behavior, CIPER, Faculdade de Motricidade Humana, Universidade de Lisboa, 1549-003 Lisboa, Portugal; cordovil.rita@gmail.com; 4 Departamento de Desporto e Saúde, Universidade de Évora, 7000-803 Évora, Portugal; gabrielasna@hotmail.com; 5 Instituto Politécnico de Viana do Castelo, Escola Superior Desporto e Lazer, 960-529 Melgaço, Portugal; lprodrigues@esdl.ipvc.pt; 6 Research Center in Sports Sciences Health Sciences and Human Development (CIDESD), 5000-801 Vila Real, Portugal

**Keywords:** motor competence assessment, childhood, high motor competence, low motor competence

## Abstract

This study examined motor competence (MC) behavior in 6- to 14-year-old children, and investigated the differences in health-related fitness (HRF) between high and low MC groups, according to sex and age. A sample of 564 children (288 males) participated in this study, divided into three age groups (6–8 years; 9–11 years; 12–14 years). Total MC and its three components (stability, locomotor, and manipulative) were assessed with a quantitative instrument. HRF was evaluated using a maximal multistage 20-m shuttle-run test and the handgrip test. Participants were divided into tertiles according to their MC level and high and low MC groups were analyzed. Overall, MC increased across age groups for both sexes, but boys presented better results than girls. The high MC group outperformed their low MC peers in all HRF variables, independent of their age group. Although cardiovascular fitness increased with age for both the high and low MC groups, the differences between these groups were greater in older children compared to younger children, within the study age range. The findings suggest that MC interventions should be considered as an important strategy to enhance HRF, and girls at a young age should be a priority target.

## 1. Introduction

The overall prevalence of childhood obesity is high [1], and children tend to spend less time and engage less in physical activity (PA) [2] while spending more time on sedentary activities [3]. A theoretical model proposed by Stodden and colleagues (2008) [4] allocates a key role to motor competence (MC) for developing an active and healthy lifestyle, and very recently, several studies provided support for this hypothetical model regarding the effects of MC on the positive developmental trajectories of health [5,6]. 

MC is used as a global term that includes a wide variety of terms used in literature (i.e., fundamental motor skill or movement, motor proficiency or performance, motor ability and motor coordination), and can be advantageously described as a person’s ability to be proficient in a wide range of motor acts or skills [7] that include locomotor, stability or manipulative movements [1,9].

The strength of the relationship between MC and health-related fitness (HRF), and MC and weight status, is high and increases with time [6]. However, MC and HRF have shown a decline over recent years [10,11,12,13] with approximately 5–8% of school aged children exhibiting motor problems often mentioned as having developmental coordination disorder (DCD) [14,15,16]. This disorder is characterized by poor MC—substantially below that expected for a child’s chronological age and measured intelligence [17]. Moreover, this poor MC renders difficult their involvement in activities of daily living, as well as sports and exercise [18], putting at risk the development of motor competence, health related fitness and health [19]. When investigating the relationship between HRF and MC, body composition can definitely be a confounder. For instance, body mass index is usually positively related to cardiorespiratory fitness [20] and MC [21], while negatively related to strength [22].

Research indicates that low MC children present lower cardiorespiratory fitness and muscle strength, two of the most important determinants of health-related fitness [23]. For example, Cairney, Hay, Faught, Flouris, and Klentrou [24] found that children with low MC (9–14 years) performed on average 17% inferior to their peers on the Progressive Aerobic Cardiovascular Endurance Run (PACER) test. Hands and Larkin [25] also found, using the same test, lower results for the less proficient group (27% lower). Similar results were found using laboratory-based methods. For example, Hands, Larkin, Parker, Straker, and Perry [26] reported a 11% difference between MC groups on cardiorespiratory endurance using the physical working capacity 170 (PWC 170) test. Moreover, longitudinal studies showed that cardiorespiratory fitness differences between motor competence groups (low vs high) tend to remain over time [27,28] or the lower MC group tends to present a much greater decline rate [29]. So, it appears to be increasingly difficult for these children to catch up with their peers. Identical results were found for groups with low MC and muscle strength [30]. In Haga’s research, the group with high MC presented higher performances (21%) at baseline and after 32 months (20%) than the group with low MC.

Emerging evidence indicates that object control/manipulative skills are more likely to be predictive of HRF in late childhood and early adolescence, while for the early stages of development, locomotor skills may have a more important role [31].

As mentioned above, in recent literature, the differences in HRF related to MC groups are very noticeable. However, and to the best of our knowledge, the instruments used did not assess all MC dimensions: locomotor, stability, and manipulative or object control.

Accordingly, the purpose of the present study was to investigate the differences, according to sex and age, in physical fitness and body composition amongst two groups with differentiated MC (i.e., higher and low MC) using a quantitative instrument [9] with good validity that provides a MC composite from its three theoretical components (i.e., locomotor, stability and manipulative skills) [8]. It is expected that the group with the highest MC group will show better results in HRF, independent of age and sex, in comparison to the group with the lowest MC [27,32].

## 2. Methods

### 2.1. Participants

A sample of 564 children (288 males) aged from 6 to 14 years old, with an average age of 10.6 years (SD = 2.40) participated in this study. Children had no motor limitations and were selected from public schools. Two physical education teachers collected all the data for this study over a period of three months during regularly scheduled classes. A local ethics committee ensured that all procedures regarding scientific research involving human beings would be conducted safely. Written informed consent was obtained from the children's parents. Children were asked for verbal assent, while being informed that participation was voluntary and that they could leave the study at any point.

### 2.2. Measures

**Health-related fitness.** HRF comprises a number of different components, such as cardiorespiratory fitness, muscle strength, muscle endurance, flexibility, and body composition [26]. In this study, three components of HRF were assessed, namely cardiorespiratory fitness (PACER), upper body strength (handgrip test) and body mass index (BMI). PACER test: This test assessed aerobic capacity by using a progressive shuttle run with an increased cadence. Participants run back and forth across a 20-m course, beginning at a slow pace with increments at every minute. The test finishes when they cannot maintain the pace at the end of two consecutive 20 m laps. The FITNESSGRAM test protocol (for more information see Welk and Meredith, [33]) was used with one modification; to ensure that the participants reached their maximum level and to give proper encouragement, an adult ran with them to pace the rhythm. Handgrip test: This test is often used for assessing muscular fitness in epidemiological studies [23]. Each participant squeezes the dynamometer with maximum isometric effort, maintained for 5 s. The best result after three attempts was recorded as the final score. Body composition: Participant’s height and weight measures were used to calculate the BMI score.

**Motor competence.** Motor competence was evaluated using the model proposed by Luz and colleagues [9], developed on a sample of Portuguese children. The model is divided into three factors/categories (stability, locomotor and manipulative) with two motor tasks each: Stability tests: (a) Shifting platforms—Moving sideways using two wooden platforms (25 cm × 25 cm × 2 cm with four 3.7 cm feet at the corners). The test begins with the participant standing on one single platform and moving the other from one side to the other (right to left or vice-versa), moving the body to the second platform, and so on for 20 s. Each successful body transfer from one platform to the other is scored with two points (one point for shifting the platform from side to side, one point for moving the body into the platform). Participants were given two trials and only the best score was considered; (b) Lateral jumps—Participants were requested to jump sideways with two feet together over a small wooden beam (60 cm length × 4 cm high × 2 cm width) located in the middle of a rectangular surface (100 cm length × 60 cm width) as fast as possible for 15 s. Each correct jump (two feet together, without touching outside the rectangle, and without stepping in the wooden beam) was scored 1 point and the best score was recorded. Locomotor tests: (a) Shuttle run (SHR)—Children were instructed to run at maximal speed between the starting line and a line placed 10 m away four times (4 × 10 m). They had to pick up a block of wood, run back and place the block beyond the starting line, then repeat it again. The task ended (time stop) when the participants crossed the starting line. The best time of the two trials was recorded; (b) Standing long jump (SLJ)—Participants had to jump with both feet simultaneously as far as possible. Participants were allowed to swing their arms back and forth. The final score was given by the longest (the best of both attempts) distance (in centimetres) between the starting line and the landing position. Manipulative tests: (a) Throwing velocity—Children were requested to throw a ball against a wall at maximum speed using an overarm action with a preparatory balance (one or two steps). A tennis ball (diameter: 6.5 cm; weight: 57 g) and a baseball (diameter: 7.3 cm; weight: 142 g) were used for children between 7 and 10 years old and for 11 years old and older, respectively. Peak velocity was measured in m/s with a velocity radar gun (e.g., Pro II Stalker radar gun). Every participant performed three trials, with the final score being the best result; (b) Kicking velocity—Children were instructed to kick a soccer ball against a wall at maximum speed using a preparatory balance (one or two steps). Three different balls were used, namely a soccer ball nº3 (circumference: 62 cm, weight: 350 g), nº4 (circumference 64 cm, weight: 360 g) and nº5 (circumference 68 cm, weight: 410 g) for children 7–8 years-old, 9–10 years-old and older than 10 years-old, respectively. Ball peak velocity was measured in m/s with a velocity radar gun (e.g., Pro II Stalker radar gun). The final result was obtained through the best performance of three attempts.

Standardized scores were calculated for each task and then stability, locomotor, and manipulative categories were calculated through the sum of the t-scores of the two representative tasks. Given the nature of the task (in sec), the SHR result-scores were inverted. As mentioned above, the MC evaluation should include tasks covering stability, locomotor and manipulative categories [8]; thus, total MC was calculated from the mean of the three categories’ outcomes (t-scores). 

All participants were evaluated in groups of five, with all five completing each test trial before the next attempt (to minimize the effects of fatigue), and in the same task order. The stability tasks were performed first, followed by the locomotor tasks and, lastly, the manipulative tasks. A proficient movement was demonstrated and one opportunity to try each task was provided to all participants. Motivational feedback was given, but the results of the tasks were not commented on.

### 2.3. Data Analysis 

All variables were delineated according to age (6–8 years; 9–11 years; 12–14 years), and sex. Health related fitness variables were furthermore divided according to MC level (tertile groups). Previous researchers used similar procedures [34,35] and because the intent was to contrast groups with high and low MC, participants with intermediate scores (i.e., middle tertile) were excluded from this analysis [26]. A multivariate analysis of covariance (MANCOVA) using pacer and handgrip strength as multivariate dependent variables was conducted for each sex separately, testing for age and MC effects on HRF and controlling for BMI. Univariate between group analyses was used to further understand the main effects results of the multivariate HRF variable, and Bonferroni post hoc pairwise comparisons were performed when needed. SPSS 20 was used for all statistical analyses with a *p* < 0.05 as the level of statistical significance.

## 3. Results

Descriptive statistics for PACER, handgrip, BMI, MC and the three components of MC for each age group, divided by sex, are displayed in Table 1.

The results revealed on average, an overall increase in MC as well as in all MC components with age development. Moreover, boys generally outperformed girls in all MC variables (except in the middle age group for the stability component).

The MANCOVA results for boys revealed a significant main effect for MC groups (Wilks’ *λ* = 0.506, *F* (2182) = 88.95, *p* < 0.001, *η*^2^*_p_* = 0.49); and for age groups (Wilks’ *λ* = 0.343, *F* (4364) = 64.40 *p* < 0.001, *η*^2^*_p_* = 0.41). Girls also displayed a significant main effect for MC groups (Wilks’ *λ* = 0.623, *F* (2175) = 52.87 *p* < 0.001, *η*^2^*_p_* = 0.38); and for age groups (Wilks’ *λ* = 0.345, *F* (4350) = 61.45 *p* < 0.001, *η*^2^*_p_* = 0.41). BMI was found to be a significant covariate in both analyses of boys and girls. 

According to the MANCOVA results, independent of BMI, the more proficient MC group consistently displayed better HRF results, and HRF results increased with age, for both sexes and MC groups. 

**Figure 1:**
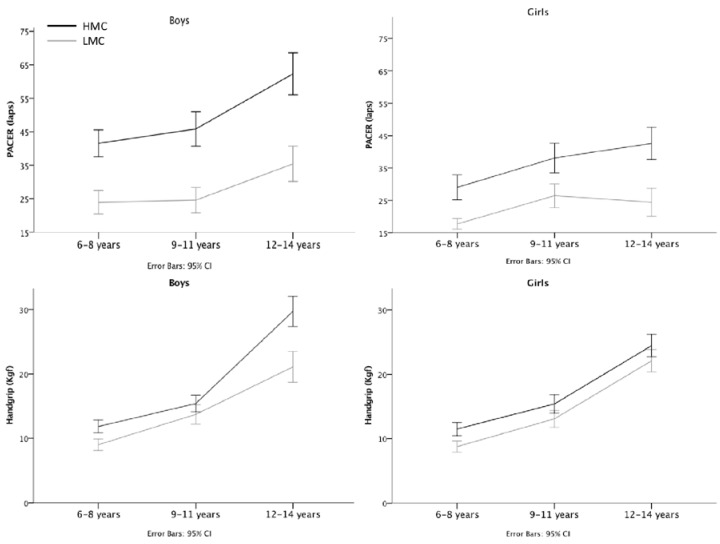
Means and 95%CI for all HRF variables in both genders. HMC—high motor competence; LMC—low motor competence.

To further clarify the results, univariate main effects for each HRF variable were examined for both sexes (see Figure 1). Boys displayed a significant main effect for the MC proficiency group for both PACER [*F* (1183) = 114.7; *p* < 0.001; *η*^2^*_p_* = 0.39], and handgrip [*F* (1183) = 86.9; *p* < 0.001; *η*^2^*_p_* = 0.32]; and significant main effects for age groups [PACER − *F* (2183) = 43.4; *p* < 0.001; *η*^2^*_p_* = 0.32; Handgrip − *F* (2183) = 149.1; *p* < 0.001; *η*^2^*_p_* = 0.62]. A significant interaction effect between age and MC groups was also found for both HRF variables [PACER − *F* (2183) = 3.1; *p* = 0.045; *η*^2^*_p_* = 0.03; Handgrip − *F* (2183) = 9.8; *p* < 0.001; *η*^2^*_p_* = 0.10]. Girls exhibited similar results to boys in the MC [PACER − *F* (1176) = 54.7; *p* < 0.001; *η*^2^*_p_* = 0.24; Handgrip − *F* (1176) = 43.7; *p* < 0.001; *η*^2^*_p_* = 0.209), and age groups main effects [PACER − *F* (2176) = 32.2; *p* < 0.001; *η*^2^*_p_* = 0.27; Handgrip − *F* (2176) = 119.8; *p* < 0.001; *η*^2^*_p_* = 0.58], but no interaction effect was found.

## 4. Discussion

In this study, we have investigated the effect of MC on the HRF of children divided into three age groups (6–8 years; 9–11 years; 12–14 years). Global results revealed that boys always outperformed girls, probably as a result of biological sex related differences and a stronger social support system and motivation towards physical activities for boys [36].

Overall MANCOVA results revealed a large effect [37] of MC levels on the multivariate HRF constituted by cardiorespiratory fitness and handgrip, regardless of weight status (BMI). Furthermore, the MC groups explained the HRF variance in about 38% for girls and 49% for boys. An effect of age was also observed, with HRF increasing across age groups, and similar explained variance (41%) for boys and girls. These results clearly show that distinct levels of motor competence are associated with distinct HRF characteristics that can be interpreted as the divergent pathways (positive and negative) described in the Stodden et al. [4] model. It is well established that children with high MC spend more time doing physical activities [38], and participating in sports [27], having more opportunities to improve their HRF. Even if our data is not longitudinal, the described effect remains regardless of age, sex, and BMI values. 

Moreover, when each HRF variable (i.e., PACER and handgrip) was analyzed, we found a significant interaction between age and MC groups in boys, meaning that MC groups tend to become more different on cardiovascular fitness and handgrip with age (Figure 1 and Table 1) in boys, while in girls the gap remained stable. Previous works found similar results for cardiovascular fitness [27,28,30], and handgrip in the younger age group [27]. Longitudinal studies confirmed that children with low MC are unlikely to catch up to their peers in time [27,28,30]. 

The novelty of this research lies in the way that MC was measured. This research used a new instrument with good validity [9] that allows the assessment of MC using all three components (i.e., locomotor, stability and manipulative skills) proposed by the Gallahue et al. [8] framework.

Longitudinal and interventional studies are needed to better understand these complex relationships and their changes across age groups. The absence of maturational information was also a limitation to our conclusions. Biological maturation influences all variables, mostly during the transition from childhood to adolescence; so, future studies should consider including an assessment of the children’s biological maturation. Although other variables (e.g., vertical jump) can be used to measure muscular strength, the handgrip task is a simple assessment method and is related to both maximal upper and lower body strength [39]. 

Although the cross-sectional design of the study makes it impossible to indicate the causality direction between MC and HRF, two very distinct HRF trajectories emerge from the age-related data analysis. This conclusion is even stronger because all results were controlled for BMI confounding effects. Body mass index is usually positively related to cardiorespiratory fitness [20] and MC [21], while negatively related to strength [22]. Using participants’ BMI as a covariate in the analysis removed the potential confounder effect of BMI, showing the true relationship between MC and HRF.

Our results lead to a conclusion that may be of paramount importance to promote the development of MC from an early age in order to decrease the possibility that children will develop negative trajectories of MC and health-related fitness, also avoiding health and social problems that derive from being overweight and obesity [32].

## Figures and Tables

**Table 1:** Means and standard deviations by age group and sex for motor competence (MC) components, health-related fitness (HRF) variables, and HRF variables according to MC groups.

Variables	6–8 Years		9–11 Years		12–14 Years	
	B *n* = 94	G *n* = 90	B *n* = 97	G *n* = 94	B *n* = 97	G *n* = 92
	M ± SD	M ± SD	M ± SD	M ± SD	M ± SD	M ± SD
Stability (pts)	43.5 ± 7.0	41.3 ± 7.1	49.2 ± 7.6	50.8 ± 8.0	59.1 ± 8.2	55.5 ± 9.1
Locomotor (pts)	46.3 ± 6.6	40.5 ± 7.9	50.7 ± 8.0	48.6 ± 7.0	61.1 ± 8.8	52.0 ± 8.3
Manipulative (pts)	46.0 ± 4.9	38.3 ± 4.8	53.7 ± 6.0	46.2 ± 4.9	64.3 ± 8.6	50.4 ± 5.6
MC total (pts)	44.6 ± 5.8	38.8 ± 6.2	51.4 ± 6.5	48.4 ± 6.0	62.9 ± 8.2	53.0 ± 7.0
BMI (kg/m^2^)	17.0 ± 1.9	17.4 ± 2.5	19.4 ± 3.7	18.7 ± 3.4	20.3 ± 3.7	21.9 ± 4.7
PACER (laps)	31.8 ± 12.6	23.1 ± 9.0	35.7 ± 16.0	30.9 ± 11.9	49.1 ± 19.0	32.8 ± 14.6
High MC	41.5 ± 10.9	29.0 ± 10.3	45.8 ± 14.3	38.1 ± 12.4	62.3 ± 17.4	42.6 ± 13.6
Low MC	23.9 ± 9.5	17.7 ± 4.4	24.6 ± 10.5	26.45 ± 9.9	35.4 ± 14.7	24.4 ± 11.6
Handgrip (Kgf)	10.4 ± 2.7	10.0 ± 2.7	14.9 ± 4.1	14.6 ± 4.1	25.3 ± 7.4	22.9 ± 4.7
High MC	11.8 ± 2.7	11.5 ± 2.8	15.4 ± 3.6	15.4 ± 3.9	29.7 ± 6.5	24.5 ± 4.8
Low MC	9.0 ± 2.5	8.7 ± 2.4	13.7 ± 4.2	13.1 ± 3.6	21.1 ± 6.6	22.1 ± 4.7

Notes: B = boys; G = girls; M = mean; SD = standard deviation; BMI—body mass index; PACER—Progressive aerobic cardiovascular endurance run.
